# Decreased head circumference at birth associated with maternal tobacco smoke exposure during pregnancy on the Japanese prospective birth cohort study

**DOI:** 10.1038/s41598-021-98311-2

**Published:** 2021-09-23

**Authors:** Tadashi Shiohama, Aya Hisada, Midori Yamamoto, Kenichi Sakurai, Rieko Takatani, Katsunori Fujii, Naoki Shimojo, Chisato Mori, the Japan Environment Children’s Study (JECS) Group, Michihiro Kamijima, Shin Yamazaki, Yukihiro Ohya, Reiko Kishi, Nobuo Yaegashi, Koichi Hashimoto, Chisato Mori, Shuichi Ito, Zentaro Yamagata, Hidekuni Inadera, Takeo Nakayama, Hiroyasu Iso, Masayuki Shima, Youichi Kurozawa, Narufumi Suganuma, Koichi Kusuhara, Takahiko Katoh

**Affiliations:** 1 Department of Pediatrics, Graduate School of Medicine, Chiba University, 1-8-1 Inohana, Chuo-ku, Chiba-shi, Chiba, 260-8670 Japan; 2 Center for Preventive Medical Sciences, Chiba University, 1-33 Yayoi-cho, Inage-ku, Chiba-shi, Chiba, 263-8522 Japan; 3 Department of Bioenvironmental Medicine, Graduate School of Medicine, Chiba University, 1-8-1 Inohana, Chuo-ku, Chiba-shi, Chiba, 260-8670 Japan; 4 Japan Environment and Children’s Study Programme Office, National Institute for Environmental Studies, 16-2 Onogawa, Tsukuba, Ibaraki 305-8506 Japan; 5 Aichi Regional Center for JECS, Nagoya City University Graduate School of Medical Sciences, Nagoya, Japan; 6 National Institute for Environmental Studies, Tsukuba, Japan; 7 National Center for Child Health and Development, Tokyo, Japan; 8 Hokkaido Regional Center for JECS, Hokkaido University, Sapporo, Japan; 9 Miyagi Regional Center for JECS, Tohoku University, Sendai, Japan; 10 Fukushima Regional Center for JECS, Fukushima Medical University, Fukushima, Japan; 11 Chiba Regional Center for JECS, Chiba University, Chiba, Japan; 12 Kanagawa Regional Center for JECS, Yokohama City University, Kanagawa, Japan; 13 Koshin Regional Center for JECS, University of Yamanashi, Yamanashi, Japan; 14 Toyama Regional Center for JECS, University of Toyama, Toyama, Japan; 15 Kyoto Regional Center for JECS, Kyoto University, Kyoto, Japan; 16 Osaka Regional Center for JECS, Osaka University, Suita, Japan; 17 Hyogo Regional Center for JECS, Hyogo College of Medicine, Hyogo, Japan; 18 Tottori Regional Center for JECS, Tottori University, Yonago, Japan; 19 Kochi Regional Center for JECS, Kochi University, Kochi, Japan; 20 University of Occupational and Environmental Health, Kitakyushu, Japan; 21 South Kyushu/Okinawa Regional Center for JECS, Kumamoto University, Kumamoto, Japan

**Keywords:** Health care, Medical research

## Abstract

Maternal tobacco smoke exposure during pregnancy impairs fetal body size, including head circumference (HC) at birth; however, the mechanism still remains unclear. This analysis using a large prospective cohort study evaluated the impact of maternal tobacco exposure on their offspring’s HC and the relationship with placental weight ratio (PWR) and placental abnormalities. Parents-children pairs (n = 84,856) were included from the 104,065 records of the Japan Environmental and Children’s Study. Maternal perinatal clinical and social information by self-administered questionnaires, offspring’s body size, and placental information were collected. Data were analyzed with binominal logistic regression analysis and path analysis. Logistic regression showed significantly elevated adjusted odds ratio (aOR) (1.653, 95% CI 1.387–1.969) for the impact of maternal smoking during pregnancy on their offspring’s smaller HC at birth. Maternal exposure to environmental tobacco smoke in the non-smoking group did not increase aOR for the smaller HC. Path analysis showed that maternal smoking during pregnancy decreased the offspring’s HC directly, but not indirectly via PWR or placental abnormalities. The quitting smoking during pregnancy group did not increase aOR for the smaller HC than the non-smoking group, suggesting that quitting smoking may reduce their offspring’s neurological impairment even after pregnancy.

## Introduction

Tobacco smoking contains an estimated 5,000 chemicals, including nicotine, and 97 other hazardous components^1,2^. Smoking is the leading cause of preventable deaths (7 million deaths by direct tobacco use and 1.2 million deaths by second-hand tobacco exposure)^3^. Nevertheless, tobacco is consumed worldwide with a worldwide mean smoking prevalence of 22.18%, despite of global efforts to control the epidemic of tobacco use^4^. Tobacco exposure during pregnancy harms maternal health and impairs fetal growth leading to a decreased body size at birth and reduced head circumference (HC)^5–10^. As well as maternal smoking, maternal exposure to environmental tobacco smoke (ETS), which is estimated prevalence of 11.1%^6^, is reported to be independently associated with smaller HC^6,10^.

Maternal smoking and second-hand smoke exposure are associated with offspring cognitive and behavioral impairments. There is increasing evidence of attentional deficits, impaired learning and memory, lowered intelligence quotient, cognitive dysfunction, and later childhood conduct problems, although not all studies have reported a significant negative relationship between maternal tobacco exposure and offspring outcomes^11,12^. Birth HC is an important physical measurement, which is easily accessed and associated with intellectual development^13–17^. Tobacco exposure during pregnancy may impair cognitive ability^18–21^ and increase the risk of attention-deficit hyperactivity disorder^22,23^, even though the results obtained so far are controversial^21^.

Maternal smoking is recognized as an unfavorable factor that causes oxidative stress in placental tissue^24^, and impairs placental development due to reducing blood flow^25^, leading to a decrease in placental weight^26,27^ and histological changes^28,29^ as well as an increased risk of placenta previa^30^, placenta abruption^31^, and miscarriage^32^. These findings suggest that maternal tobacco exposure during pregnancy causes decreased brain size at birth due to placental impairment. However, the evidence on the relationship of prenatal smoking exposure with placental impairment and decreased HC remains unclear in prospective birth cohort studies^5,7^ and retrospective birth cohort studies^6,8^. We assessed the hypothesis that prenatal smoking leads to a smaller HC due to placental dysfunctions using a prospective large birth cohort. In addition, we analyzed the association of maternal tobacco exposure during pregnancy and HC at birth with potential covariants, including placental information.

## Materials and methods

### Study design and participants

The data set in this study was adopted from the Japan Environmental and Children’s Study (JECS). JECS is an ongoing prospective birth cohort study, which was a national project funded directly by the Ministry of Environment to elucidate the influence of environmental factors during the fetal period and early childhood with follow-up until 13 years old. The protocol and baseline data of the JECS were published elsewhere^33–35^. The JECS protocol was approved by the Ministry of the Environment’s Institutional Review Board on Epidemiological Studies and the ethics committees of all participating institutions; the Medical Support Centre (National Centre for Child Health and Development), and 15 Regional Centers (Hokkaido University, Tohoku University, Fukushima Medical University, Chiba University, Yokohama City University, University of Yamanashi, University of Toyama, Nagoya City University, Kyoto University, Osaka University, Hyogo College of Medicine, Tottori University, Kochi University, University of Occupational and Environmental Health, and Kumamoto University). The JECS was conducted in accordance with the Declaration of Helsinki and other internationally valid regulations and guidelines, and with written informed consent from all participants.

Pregnant women were enrolled between January 2011 and March 2014 under the following inclusion criteria: (1) being resident in any of the 15 Study Areas at the time of recruitment and enrolled with Co-operating health care providers; (2) having an expected delivery date after August 1, 2011; and (3) being capable of comprehending the Japanese language and completing the self-administered questionnaire.

**Figure 1:**
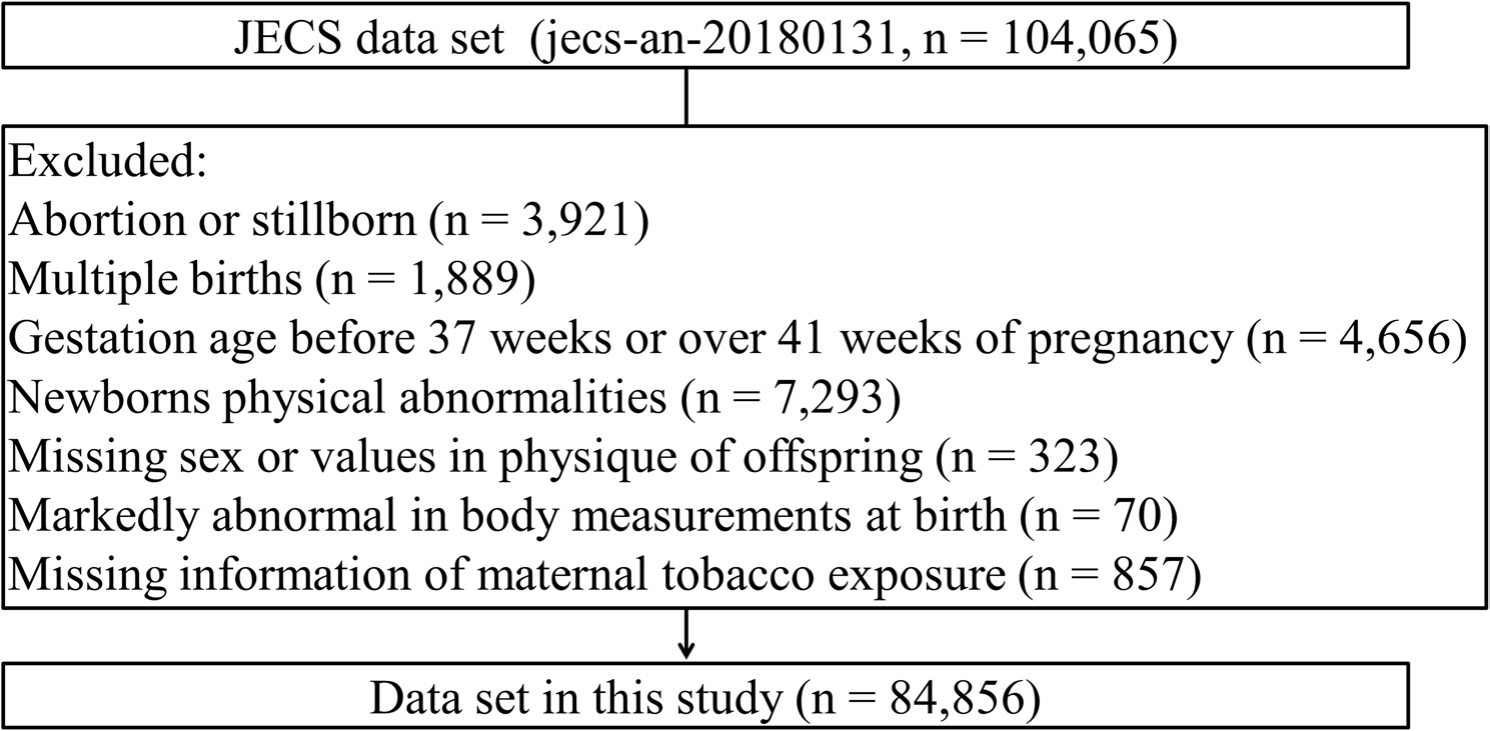
Flowchart of participant selection from the Japan Environment and Children’s Study (JECS) birth cohort.

The current study employed the jecs-an-20180131 data set, which was released in March 2018. This data set included 104,065 fetal records. From these 104,065 records, we excluded cases with abortion or stillborn babies (n = 3,921), with multiple births (n = 1,889), with a gestational age before 37 weeks or over 41 weeks of pregnancy (n = 4,656), with newborns physical abnormalities (n = 7,293), with markedly abnormal body measurements at birth (n = 70), and with missing information about sex and body measurements at birth (n = 323) or maternal tobacco exposure (n = 857). Newborns with physical measurements outside the following ranges were excluded from statistical analyses: HC of 20–50 cm, body weight over 1000 g, body height over 30 cm, and chest circumference 20–40 cm. In total, 84,856 parents-children pairs were included in our analyses (Fig. 1).

### Data collections

The maternal age, maternal height, maternal body mass index (BMI) (body weight [kg]/height [m]^2^), maternal parity, gestational hypertension, diabetes mellitus/gestational diabetes mellitus, and placental information were collected from medical records transcripts. Maternal education status, household income, maternal smoking, maternal exposure to ETS, and maternal alcohol exposure were collected by self-administered questionnaires at mid-late pregnancy. The information on maternal smoking exposure was categorized to 1 = never smoked (Never), 2 = quit before pregnancy (Previously did, but quit before recognizing current pregnancy), 3 = quit during pregnancy (Previously did, but quit after finding out current pregnancy), and 4 = smoking during pregnancy (Yes, I still smoke). The information on maternal exposure to ETS was categorized to 1 = seldom, 2 = 1–3 times a week, 3 = 4–7 times a week.

Offspring’s body size at birth was assessed according to the Japanese neonatal anthropometric charts for gestational age at birth^36^. According to a previous epidemiological study^37^, the point of smaller HC was determined as HC < 3rd percentile calculated by sex and gestational age in the Japanese standard reference^36^.

### Statistical analysis

Clinical and social characteristics of parent–child participants for each sex were compared using Mann–Whitney U test and chi-squared tests. The outcome variables were cases of smaller HC, and placental abnormalities (placenta previa, abruption placentae, placental calcification, placental infarction, and other placental abnormalities were identified using medical records transcripts). Other placental abnormalities included nuchal cord, low-lying placenta, velamentous insertion, hyper coiled cord, excessively long umbilical cord, multilobate placenta, succenturiate placenta and placenta accreta. The effects of parental smoking and maternal exposure to ETS on smaller HC or placental abnormalities were evaluated as an odds ratio (OR) or adjusted odds ratio (aOR), and 95% confidence interval (CI) using binomial logistic regression analysis with crude and adjusted models. We conducted adjustments for maternal age, height, pre-pregnancy BMI and parity, maternal alcohol drinking during the first to third trimesters, placental weight (g) to birth weight placental weight (g) ratio (placental weight ratio, PWR) which is used to indicate the adequacy of fetal nutrition^38^, and gestational age.

The relationship of maternal smoking exposure with the offspring’s HC, placental weight, PWR, and the presence of placental anomalies were analyzed with path analysis. For missing values estimation, we used full information maximum likelihood estimation. Model fit was assessed using the root mean square error of approximation (RMSEA) ≤ 0.05 and comparative fit index (CFI) > 0.95. P < 0.05 was considered significant. Statistical analyses were performed using IBM SPSS Statistics version 23 (IBM Corp. Armonk, NY). Outliers of physical measurements were evaluated with Smirnov‐Grubbs test using R version 3.6.3^39^.

## Results

This study enrolled 84,856 parents-child pairs after selection (Fig. 1). The range of outliers generated using the offsprings’ physical measurements as exclusion criteria were similar to those calculated by Smirnov–Grubbs’ test (HC ≤ 24 cm or ≥ 43 cm, body weight ≤ 540 g or ≥ 4890 g, body height ≤ 39.5 cm, and chest circumference ≤ 24 cm or ≥ 39.5 cm).

Comparing the male group’s and the female group’s backgrounds showed no significant differences in maternal body size, parental smoking status, maternal educational status, and household income (Table 1). There were significant differences in offspring body size and placental information between the male and female offspring (Table 1).

**Table 1:** Clinical and social characteristics of participants.

	Total (n = 84,856)	Male offspring (n = 43,042)	Female offspring (n = 41,814)	*p*-value
Maternal age (years old)	31 (28, 35)	31 (28, 35)	31 (28, 35)	0.256 ^a^
Maternal length (cm)	158 (154, 162)	158 (154, 162)	158 (154, 162)	0.576^a^
Pre-pregnancy BMI (Kg/m^2^)	20.5 (19.1, 22.5)	20.5 (19.1, 22.5)	20.5 (19.1, 22.5)	0.867^a^
**Parity**				0.887^b^
0	33,117 (39.9%)	16,767 (39.9%)	16,350 (40%)	
1	32,570 (39.3%)	16,561 (39.4%)	16,009 (39.2%)	
2	13,607 (16.4%)	6,892 (16.4%)	6,715 (16.4%)	
≥ 3	3,625 (4.4%)	1,822 (4.3%)	1,803 (4.4%)	
**Maternal smoking**				0.932^b^
Never smoked	48,783 (57.5%)	24,770 (57.5%)	24,013 (57.4%)	
Quit before pregnancy	20,357 (24%)	10,302 (23.9%)	10,055 (24%)	
Quit during pregnancy	11,751 (13.8%)	5,945 (13.8%)	5,806 (13.9%)	
Smoking during pregnancy	3,965 (4.7%)	2,025 (4.7%)	1,940 (4.6%)	
**Maternal exposure to ETS**				0.652^b^
Seldom	52,141 (62.1%)	26,507 (62.2%)	25,634 (61.9%)	
1–3 times a week	17,001 (20.2%)	8,587 (20.2%)	8,414 (20.3%)	
4–7 times a week	14,875 (17.7%)	7,513 (17.6%)	7,362 (17.8%)	
**Maternal alcohol drinking**				0.575^b^
Never drank alcohol	28,366 (33.4%)	14,404 (33.5%)	13,962 (33.4%)	
Quit before pregnancy	15,045 (17.7%)	7,574 (17.6%)	7,471 (17.9%)	
Drinking alcohol during the first to third trimester	41,406 (48.8%)	21,048 (48.9%)	20,358 (48.7%)	
**Education status of mother**				0.100^b^
< 10 years	4,043 (4.8%)	2,075 (4.9%)	1,968 (4.8%)	
10–12 years	26,504 (31.6%)	13,304 (31.3%)	13,200 (32%)	
13–16 years	51,995 (62.1%)	26,454 (62.3%)	25,541 (61.9%)	
≥ 17 years	1,224 (1.5%)	647 (1.5%)	577 (1.4%)	
**Household income**				0.114^b^
< 2,000,000 yen	4,429 (5.7%)	2,304 (5.8%)	2,125 (5.5%)	
2,000,000–4,000,000	27,107 (34.7%)	13,636 (34.4%)	13,471 (34.9%)	
4,000,000–6,000,000	25,795 (33%)	13,132 (33.2%)	12,663 (32.8%)	
6,000,000–8,000,000	12,450 (15.9%)	6,207 (15.7%)	6,243 (16.2%)	
8,000,000–10,000,000	5,156 (6.6%)	2,629 (6.6%)	2,527 (6.5%)	
≥ 10,000,000	3,284 (4.2%)	1,677 (4.2%)	1,607 (4.2%)	
Gestational hypertension	2,218 (2.6%)	1,097 (2.5%)	1,121 (2.7%)	0.227^b^
Diabetes or gestational diabetes mellitus	2,539 (3%)	1,305 (3%)	1,234 (3%)	0.490^b^
**Gestational age (day)**	**277 (270, 282)**	**276 (270, 282)**	**277 (271, 283)**	** < 0.001**^**a**^
**Birth weight (g)**	**3050 (2816, 3296)**	**3096 (2864, 3344)**	**3002 (2776, 3244)**	** < 0.001**^**a**^
**Birth length (cm)**	**49.0 (48.0, 50.4)**	**49.5 (48.0, 50.6)**	**49.0 (47.6, 50.0)**	** < 0.001**^**a**^
**Head circumference (cm)**	**33.2 (32.5, 34.0)**	**33.5 (32.5, 34.5)**	**33.0 (32.0, 34.0)**	** < 0.001**^**a**^
**Placenta weight (g)**	**550 (490, 625)**	**556 (500, 630)**	**550 (490, 620)**	** < 0.001**^**a**^
**Placental weight ratio**	**0.18 (0.17, 0.20)**	**0.18 (0.16, 0.20)**	**0.18 (0.17, 0.20)**	** < 0.001**^**a**^
**Placental abnormalities**	**18,895 (22.6%)**	**10,063 (23.7%)**	**8,832 (21.4%)**	** < 0.001**^**a**^
Placenta Previa	366 (0.4%)	186 (0.4%)	180 (0.4%)	0.971^b^
Abruption placentae	218 (0.3%)	116 (0.3%)	102 (0.2%)	0.462^b^
Calcification	3,349 (4%)	1,746 (4.1%)	1,603 (3.9%)	0.095^b^
**Placental infarction**	**1,869 (2.2%)**	**1,003 (2.4%)**	**866 (2.1%)**	**0.010**^**b**^
**Other placental abnormalities**	**14,134 (16.7%)**	**7,562 (17.6%)**	**6,572 (15.7%)**	** < 0.001**^**b**^

^a^Mann–Whitney U Test, ^b^chi-square test. The values are shown as median (IQR) for continuous variables or the number (percent) for nominal variables. Bold numbers indicate values with statistical significance.

Adjusted binominal logistic regression analysis of maternal tobacco exposure for predicting the offsprings’ smaller HC showed a significantly increased aOR for smaller HC only by maternal smoking during pregnancy in the offspring (aOR 1.653, CI 1.387–1.969) (Table 2). Maternal exposure to ETS did not significantly increase aOR for smaller HC in offsprings with “never smoked” mothers (Table 2). Additionally, number of daily cigarettes did not significantly increase aOR for smaller HC in offsprings with “smoking during pregnancy” mothers (Table 2).

**Table 2:** Binominal logistic regression analysis of maternal smoking and maternal exposure to ETS for predicting offspring’s smaller circumference.

**Total offsprings (n = 84,856)**			
Maternal smoking^a^			
Crude model		Adjusted model^c^	
1	1.000 (reference)	1	1.000 (reference)
2	0.894 (0.812–0.985)	2	0.988 (0.892–1.094)
3	1.032 (0.922–1.156)	3	0.982 (0.868–1.110)
4	**1.513 (1.296–1.766)**	4	**1.653 (1.387–1.969)**
Maternal exposure to ETS^b^			
Crude model		Adjusted model^c^	
1	1.000 (reference)	1	1.000 (reference)
2	**1.107 (1.004–1.22)**	2	1.066 (0.963–1.181)
3	**1.197 (1.083–1.322)**	3	1.091 (0.973–1.222)
**The offsprings of mothers with “never smoked” (n = 48,381)**			
Maternal exposure to ETS^c^			
Crude model		Adjusted model^d^	
1	1.000 (reference)	1	1.000 (reference)
2	1.048 (0.918–1.195)	2	1.017 (0.887–1.166)
3	1.069 (0.908–1.258)	3	1.039 (0.876–1.232)
**The offsprings of mothers with “smoking during pregnancy” (n = 3,953)**			
Number of daily cigarettes			
Crude model		Adjusted model^c^	
< 5	1.000 (reference)	< 5	1.000 (reference)
5–9	0.781 (0.503–1.212)	5–9	0.780 (0.496–1.227)
10–14	0.838 (0.549–1.279)	10–14	0.933 (0.602–1.445)
15–19	0.939 (0.535–1.646)	15–19	1.114 (0.627–1.982)
> = 20	0.816 (0.466–1.430)	> = 20	0.927 (0.517–1.661)

Binominal logistic regression analysis was constructed to evaluate parental smoking for predicting their offspring’s smaller head circumference (HC) (small HC was defined as less than three percentile for their gestational age and sex) in the total offsprings (n = 84,856), in the offsprings of mothers with “never smoked” (n = 48,381), and in the offsprings of mothers with “smoking during pregnancy” (n = 3,953). The values are shown as odds ratios (95% confidence interval). Bold, increasing or decreasing tendency of offspring’s smaller head circumference risk.

^a^1 = never smoked, 2 = quit before pregnancy, 3 = quit during pregnancy, 4 = smoking during pregnancy.

^b^1 = seldom, 2 = 1–3 times a week, 3 = 4–7 times a week.

^c^Adjusted for maternal age, height, pre-pregnancy BMI and parity, maternal alcohol drinking during first to third trimesters, placental weight ratio, and gestational age.

We performed adjusted binomial logistic regression analysis of parental smoking for predicting placenta previa, abruption placentae, placental calcification, and placental infarction in total offsprings (Table 3). Maternal smoking during pregnancy increased the aOR of placental calcification and infarction. Especially, placental calcification showed a clear dose–response with maternal smoking. On the other hand, maternal exposure to ETS did not elevate the risk of these placental abnormalities (Table 3).

**Table 3:** Adjusted binominal logistic regression analysis of parental smoking for predicting placental abnormalities.

	Placenta previa aOR (95%CI)	Abruptio placentae aOR (95% CI)	Placental calcification aOR (95% CI)	Placental infarction aOR (95% CI)
**Maternal smoking**^**a**^				
1	1.000 (reference)	1.000 (reference)	1.000 (reference)	1.000 (reference)
2	1.183 (0.924–1.516)	0.759 (0.524–1.100)	0.888 (0.810–0.973)	0.956 (0.851–1.074)
3	0.988 (0.692–1.411)	1.188 (0.787–1.794)	**1.126 (1.012–1.253)**	0.893 (0.767–1.040)
4	0.780 (0.420–1.449)	1.547 (0.854–2.802)	**1.691 (1.449–1.973)**	**1.352 (1.083–1.688)**
**Maternal exposure to ETS**^**b**^				
1	1.000 (reference)	1.000 (reference)	1.000 (reference)	1.000 (reference)
2	1.014 (0.769–1.337)	0.992 (0.690–1.426)	1.077 (0.983–1.180)	1.036 (0.919–1.168)
3	0.993 (0.722–1.367)	0.978 (0.652–1.466)	1.104 (0.998–1.221)	1.005 (0.876–1.154)

Binominal logistic regression analysis was constructed to evaluate parental smoking for predicting placenta previa, abruption placentae, placental calcification, and placental infarction. The values are shown as adjusted odds ratios [aOR] (a 95% confidence interval [95% CI]). Bold, increasing or decreasing tendency of offspring’s smaller head circumference risk.

^a^1 = never smoked, 2 = quit before pregnancy, 3 = quit during pregnancy, and 4 = smoking during pregnancy.

^b^1 = seldom, 2 = 1–3 times a week, 3 = 4–7 times a week.

Adjusted for maternal age, pre-pregnancy BMI and parity, maternal drinking during first to third trimesters, placental weight ratio, and gestational age.

**Figure 2:**
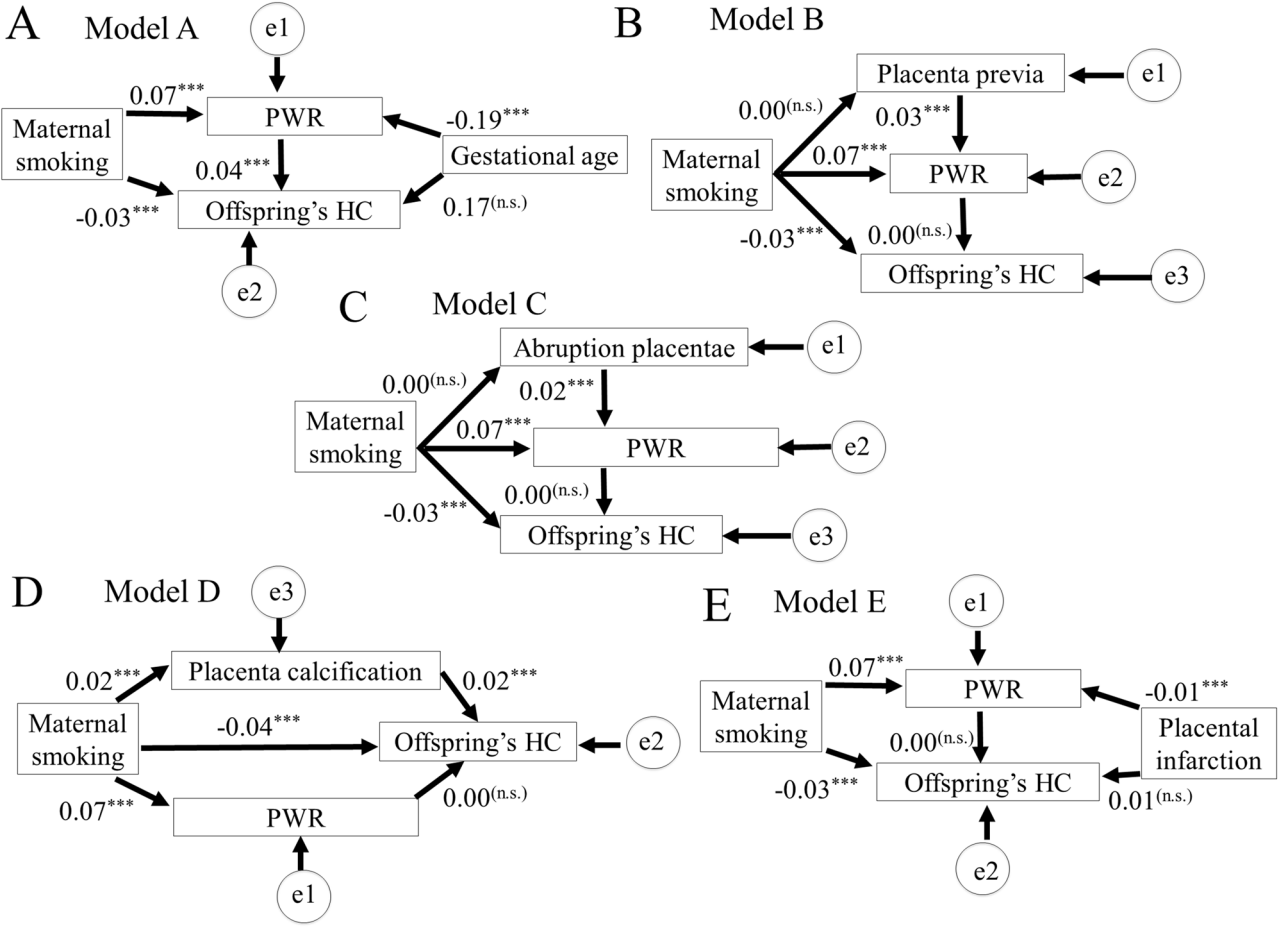
Path analysis for the relationship between maternal smoking toward offspring’s HC, PWR and placental abnormalities (**A**, gestation age; **B**, placenta previa; **C**, abruption placentae; **D**, placental calcification; **E**, placental infarction) showing the standardized estimates with p-values and coefficient of determination (*R*^2^). *: *p* < 0.05, **: *p* < 0.01, ***: *p* < 0.001, (n.s.): not significant. ”e” represent the errors. HC, head circumference; PWR, placental weight ratio.

To evaluate the relationship among maternal smoking, PWR, placental abnormalities, and offspring’s HC, we performed path analysis using path analysis in total offsprings (Fig. 2, Tables 4 and 5). We found that maternal smoking during pregnancy had counteract effect on offspring’s HC at birth. Path analyses showed that maternal smoking directly decreased their offspring’s HC (patho = − 0.027) (Fig. 2A, Tables 4 and 5), but did not indirectly change their offspring’s HC due to placenta previa (Fig. 2B, Tables 4 and 5), abruption placentae (Fig. 2C, Tables 4 and 5), placental calcification (Fig. 2D, Tables 4 and 5) or placental infarction (Fig. 2E, Tables 4 and 5), though R2 for offspring’s HC is low (0.001–0.03). We also tried to include alcohole drinking during pregnancy in the path analysis; however, we could not find a significant model with the covariate.

**Table 4:** Direct and indirect effects and intermediate rate of predictor variables.

Pathways		Standardized coefficients
Model A	Maternal smoking → Offspring’s HC (direct effect)	− 0.027
	Maternal smoking → PWR → Offspring’s HC (indirect effect)	0.003
Model B	Maternal smoking → Offspring’s HC (direct effect)	− 0.025
	Maternal smoking → PWR → Offspring’s HC (indirect effect)	0
Model C	Maternal smoking → Offspring’s HC (direct effect)	− 0.025
	Maternal smoking → PWR → Offspring’s HC (indirect effect)	0
Model D	Maternal smoking → Offspring’s HC (direct effect)	− 0.026
	Maternal smoking → PWR → Offspring’s HC (indirect effect)	0.001
Model E	Maternal smoking → Offspring’s HC (direct effect)	− 0.025
	Maternal smoking → PWR → Offspring’s HC (indirect effect)	0

Each model is assigned for the Fig. 2 panel with same letter (e.g. Model A is assigned for Fig. 2A).

*HC* head circumference, *PWR* placental weight ratio.

**Table 5:** Model Fit Summary for the Final Path Analysis.

Index	Criterion for good fit	Mode A	Model B	Model C	Model D	Model E
χ^2^ (df = 1)	/	1.309	0.075	5.856	12.975	0.676
χ^2^ p value	> 0.05	0.252	0.758	0.016	< 0.001	0.411
CFI	> 0.95	1.000	1.000	0.990	0.977	1.000
RMSEA	< 0.05	0.002	< 0.001	0.008	0.012	< 0.001
R^2^ for PWR	/	0.040	0.006	0.005	0.005	0.005
R^2^ for offsprings’ HC	/	0.029	0.001	0.001	0.001	0.001

Each model is assigned for the Fig. 2 panel with same letter (e.g. Model A is assigned for Fig. 2A).

*CFI* comparative fit index, *HC* head circumference, *PWR* placental weight ratio, *RMSEA* root mean square error of approximation.

## Discussion

We performed logistic regression analysis and path analysis on this cohort birth study to evaluate the impact of parental smoking on their offspring’s HC at birth. Our results showed that maternal smoking during pregnancy directly increased the risk of reduced HC, which was independent of PWR or placental abnormalities.

There have so far been four large cohort studies with over 10,000 participants to evaluate the impact of parental smoking on offspring’s HC^5–8^. Our study was the largest-scale study with over 80,000 participants enrolled. A birth cohort study on the Murmansk County Birth Registry in Russia^5^ reported that maternal smoking increased the risk of reduced HC with an aOR of 1.69 in the 1–5 cigarettes per day, 2.08 in the 5–10 cigarettes per day, and 5.19 in the over ten cigarettes per day groups. Single institutional birth cohorts reported that maternal smoking during pregnancy was associated with HC with an aOR of 1.06^6^ and 1.58^7^. Inoue et al. reported that in non-smoking pregnant women, environmental tobacco exposure was associated with a − 0.24 cm difference in HC^8^. Meta-analysis studies found maternal active smoking was associated to a smaller HC at birth^9,10^. The decreased HC by parental smoking exposure was also observed during the fetal period by echographic examination^40–43^.

Previous reports found that placental weight was positively associated with HC^44–46^, while some investigators reported that maternal smoking was associated with decreased placental weight^26,27^. The present study found that maternal smoking was associated with a smaller HC, and a study by Mitsuda et al. using the same dataset found that maternal smoking was associated with increased placental weight^47^. Using path analysis, we found that maternal smoking was directly associated with a smaller HC, but was not indirectly associated with a smaller HC via PWR or placental abnormalities.

The pathophysiological mechanism of how maternal smoking led to a smaller HC, not due to placental weight or placental abnormalities, remains unclear. The possible serious confounders of a negative effect of maternal smoking in infantile brain development in such an investigation are innumerable^48^. We speculated the specific negative effect of maternal smoking on offsprings’ HC could be related to a positive association between maternal smoking and premature closure of one or more of the cranial sutures^49^. There was a possibility that our cohort involved newborns with premature closure of the cranial sutures, even although we excluded offsprings with physical abnormalities.

From another viewpoint, maternal smoking in late pregnancy causes in utero hypoxia and placental insufficiency, which causes the placenta to grow relative to the growing fetus as a compensatory response to provide sufficient oxygen and nutrients to the fetus. Therefore, enhanced angiogenesis and increased development of new vessels are observed in the placentas of women who continued smoking throughout pregnancy^50^. Maternal smoking during pregnancy was higher BMI and gained more weight during pregnancy, and these factors are also associated with the heavy placenta^51^. These pathophysiological changes in the placentas may explain intrauterine growth restriction and compensatory normalization of PWR that occurred in women who smoked throughout pregnancy.

Importantly, the group that quit smoking during pregnancy did not have a significantly higher risk of their offspring having a small HC compared to the non-smoking group (Table 2), suggesting that quitting smoking even after pregnancy potentially reduces their offspring’s neurological impairment.

Shobeiri et al. reported that maternal smoking increased the risk of abruption placentae or placenta previa^30,31^. In our statistical analyses, maternal smoking increased aOR of placental calcification and infarction, while it did not increase aOR of placental previa or abruptio placentae (Table 3). Additionally, maternal exposure to ETS did not increase aOR of these placental abnormalities (Table 3).

MRI-based analyses showed that decreased HC was strongly associated to decreased brain volumes at least in young children^52,53^, and several studies reported the association between HC and risk of intellectual disability, autism spectrum disorder, and attention-deficit hyperactivity disorder^13–17^. HC at birth has a positive relationship with higher intelligence quotient^13,15^, lower risk of attention-deficit hyperactivity disorder^14^, and neurocognitive disorder^16^. Hence, HC at birth is a useful neurological biomarker.

Our birth cohort study secondly found the smoking prevalence among Japanese women of reproductive age. In our study, 57% of 85,059 mothers selected “never smoked” in the self-administered questionnaires. The smoking prevalence among Japanese women aged 20–39 in the 2010s has been reported 6.8–16.9%^54^ and 16.5%^55^. The smoking prevalence reported by previous studies reflected the current smoking status of women of reproductive age, while our study showed that the prevalence of experienced smoker among Japanese women of reproductive age was higher than 40%. The difference of prevalence values may suggest the young women have many smoking opportunities than expected.

This study has some limitations. First, our exclusion criteria for remarked abnormal in body size at birth was determined according to our original exclusion criteria, not to previous epidemiological methodological studies, because this study is aimed to evaluate risk factor for small HC. At least, the range of outliers generated using the offsprings’ physical measurements as exclusion criteria were similar to those calculated by Smirnov-Grubbs’ test.

Second, intrauterine tobacco exposure was assessed by self-administered questionnaires and not assessed with chemical biomarkers. We could not completely rule out the possibility that mothers respond negatively and incorrectly to answer to “did you smoke during pregnancy?” out of shame or knowing the harm to fetal health of smoking during pregnancy. Using self-reports may have introduced misclassification mainly due to underreporting of cigarette consumption, which could lead to underestimation of the effects. Although the concentration of carbon monoxide, thiocyanate, or nicotine metabolites as cotinine (especially in meconium)^56^ are potential biomarkers for tobacco exposure, these have a short half-life. The ideal chemical biomarker for parental tobacco exposure has not yet been established^43,57^.

Finally, our study did not include a neurodevelopmental test or neuroimaging examination. Quantitative brain morphometry using brain magnetic resonance imaging would contribute to a more detailed and quantitative analysis of the associations between intrauterine tobacco exposure and brain morphology. There are a few possibilities of the presence of undetected brain ischemic changes in infants.

In conclusion, our statistical analyses on a large birth cohort data revealed that maternal smoking during pregnancy decreased offspring’s HC independent of placental weight changes or placental abnormalities.

## Abbreviations

CI: Confidence interval
CFI: Comparative fit index
EST: Environmental tobacco smoke
HC: Head circumference
JECS: The Japan Environment and Children’s Study
OR: Odds ratio
PWR: Placental weight ratio
RMSEA: Root mean square error of approximation
